# Supplementary material for: Sampling site for SARS-CoV-2 RT-PCR—An intrapatient four-site comparison from Tampere, Finland
Source: PLoS One. 2021 Nov 16;16(11):e0260184. doi: 10.1371/journal.pone.0260184 (PMC8594827; doi:10.1371/journal.pone.0260184)
Supplement: Attachment — Submitted filename: Response to Reviewers.docx [file pone.0260184.s001.docx]

# Response to reviewers:

## Reviewer #1:

We have added a paragraph about sample size limitation in the text, starting from line 134.

We have changed all the phrases “no difference” to “no statistically significant difference”.

We have removed lines originally at 129 to 131.

## Reviewer #2:

Regarding the question about a possible error at line originally at 125. Yes, it should read “higher” rather than “lower” and it is now corrected.

We have added a paragraph about sample size limitation in the text, starting from line 134. In addition, we have added a mention in the discussion about the patients being symptomatic and of relatively high viral load.

We have changed the conclusion sentence to emphasize the symptomatic patients, line 116.

## Reviewer #3:

We have added a paragraph about sample size limitation in the text, starting from line 134.

We have added sentence about the inclusion symptoms for the patients to the manuscript, line 51. The patients had to have had upper respiratory symptoms including loss of smell.

Two different testing swabs for the oropharynx and anterior nasal samples were used. These swabs were macroscopically identical flocked nylon swabs with a thicker handle compared to the nasopharyngeal swabs, for ergonomic and safety reasons. Both are from very reputable manufacturers, and we use these swabs from both manufacturers for PCR samples at our lab successfully so we felt that it would have been extraneous, within the scope of this study, to first asses their real-world performance on patients.

We have added text about the target genes, line 87.

The Ct values of each target gene were pooled to a mean Ct value per sample to enable comparison between patients, as a sample can be positive with one, two or three genes. The Ct value deviations between different genes within the sample were also small.

# Amended funding statement:

This work was supported by funding from Tampere Tuberculosis Foundation; Research Foundation of the Pulmonary Diseases; Competitive State Research Financing of the Expert Responsibility area of Tampere University Hospital; Tampere University Hospital Support Foundation. Funders did not have any role in any part of the study. The funders had no role in study design, data collection and analysis, decision to publish, or preparation of the manuscript.
